# Is neutrophil gelatinase-associated lipocalin unaffected by convective continuous renal replacement therapy? Definitely … maybe

**DOI:** 10.1186/s13054-015-1104-5

**Published:** 2015-11-17

**Authors:** Patrick M. Honore, Rita Jacobs, Inne Hendrickx, Elisabeth De Waele, Viola Van Gorp, Herbert D. Spapen

**Affiliations:** ICU Department, Universitair Ziekenhuis Brussel, Vrije Universiteit Brussel, 101 Laarbeeklaan, 1090 Jette Brussels, Belgium

Two recent studies published in *Critical Care* reported that plasma [1] and urinary [2] levels of neutrophil gelatinase-associated lipocalin (NGAL), an important biomarker for prediction and diagnosis of acute kidney injury, were not affected by continuous renal replacement therapy (CRRT).

The investigators assessed NGAL elimination during continuous venovenous hemofiltration [1] and hemodiafiltration [2] using respectively a cellulose triacetate [1] and a polysulfone [2] membrane filter. Of note is that these filters both have notoriously low adsorption capacity [3]. Recently, the proinflammatory high-mobility group box 1 protein, a cytokine with a molecular weight approximating that of NGAL, was also found to be unaffected by convective CRRT. However, it was significantly (up to 90 %!) cleared from the circulation when highly adsorptive membranes (i.e., surface-treated acrylonitrile 69 and polymethylmethacrylate) were used [4].

These membranes are increasingly applied for hemofiltration in critically ill patients [4]. Thus, it is imperative to evaluate NGAL clearance during convective CRRT performed with highly adsorptive membranes before definitively accepting that CRRT leaves the sensitivity of this biomarker intact.

## Abbreviations

CRRT: Continuous renal replacement therapy
NGAL: Neutrophil gelatinase-associated lipocalin
